# Plateletcrit and Mean Platelet Volume in the Evaluation of Alcoholic Liver Cirrhosis and Nonalcoholic Fatty Liver Disease Patients

**DOI:** 10.1155/2021/8867985

**Published:** 2021-02-15

**Authors:** Agata Michalak, Halina Cichoż-Lach, Małgorzata Guz, Joanna Kozicka, Marek Cybulski, Witold Jeleniewicz

**Affiliations:** ^1^Department of Gastroenterology with Endoscopy Unit, Medical University of Lublin, Jaczewskiego 8, 20-954 Lublin, Poland; ^2^Department of Biochemistry and Molecular Biology, Medical University of Lublin, Chodźki 1, 20-093 Lublin, Poland

## Abstract

Platelet (PLT) indices have been proposed as potential markers in the assessment of liver fibrosis and exacerbation of liver failure. The aim of our study was to verify mean platelet volume (MPV), platelet distribution width (PDW), and plateletcrit (PCT) in alcohol-related liver cirrhosis (ALC) and nonalcoholic fatty liver disease (NAFLD) patients. One hundred forty-two patients with ALC, 92 with NAFLD, and 68 in control group were enrolled in this study. Hematological indices (MPV, PCT, and PDW) and serological (indirect and direct) markers of liver fibrosis (AAR, APRI, FIB-4, GPR, PICP, PIIINP, TGF-*α*, PDGF-AB, laminin) were measured in each participant. MELD score in ALC patients and NAFLD fibrosis score (NFS) together with BARD score in the NAFLD group were also obtained. Results were compared between research and control groups. Then, a correlation between evaluated indices was performed in study groups. Receiver operating characteristic curves (ROCs) and area under the curve (AUC) values were applied to assess the diagnostic accuracy of measured indices. Significant increase in PDW and decrease in PCT in comparison to controls were noted in examined ALC (60.4% vs. 51.2% and 0.1% vs. 0.21%, respectively, *p* < 0.0001) and NAFLD (54.75% vs. 51.2% and 0.19 vs. 0.21%, respectively, *p* < 0.01) patients. Decreased level of MPV was observed in NAFLD group (7.85 fl vs. 8.90 fl, *p* < 0.0001). Additionally, PCT correlated with NFS (*p* < 0.0001). Evaluated PLT indices correlated with MELD score (MPV and PDW, *p* < 0.001; PCT, *p* < 0.05). They correlated with indirect and direct markers of liver fibrosis in the whole research group, too. PCT was the parameter with the greatest diagnostic accuracy in ALC patients (AUC = 0,839 for cutoff < 0.17%); in NAFLD group, it was MPV (AUC = 0,808 for cutoff < 7.9 fl). PCT in ALC and MPV in NAFLD can be perceived as potential diagnostic markers.

## 1. Introduction

Liver cirrhosis (LC), being a final stage of diverse chronic liver diseases (CLDs), constitutes a severe systemic condition with a broad range of life-threatening complications. Its manifestation usually remains asymptomatic until the decompensation of the disease, when a fibrotic cascade in hepatocytes cannot be reversed. Alcohol-related liver disease (ALD) and nonalcoholic fatty liver disease (NAFLD) belong to the most common hepatic pathologies with a global burden. According to international data, ALD is responsible for more than one million deaths yearly, and NAFLD might concern nowadays even 35% humans all over the world. From a clinical point of view, an early diagnosis of fibrotic rebuilding within the liver is of crucial importance, because it improves the outcome of patients diagnosed with CLDs [1–4]. Liver biopsy is still commonly described as a gold standard in the evaluation of fibrosis. Nevertheless, this method is connected with several significant limitations, e. g., the assessment only of a tiny part of the liver during a single procedure, verification of biopsy specimen dependent from the pathologist, and finally, serious possible complications (internal bleeding, perforation of biliary tract). The invention of ultrasound and magnetic resonance elastography turned out to be an essential progress in the imaging assessment of liver fibrosis. Their high diagnostic accuracy has been recently emphasized especially among patients with LC due to hepatitis B and C virus (HBV and HCV). However, these diagnostic tools are available only in selected medical centers due to economic reasons, and they are not used commonly in everyday clinical practice. Thus, there is still a great demand on new, commonly accessible methods in the evaluation of liver fibrosis [5–7]. Alcohol-related liver cirrhosis (ALC) and NAFLD emerge as an important global burden, and a precise noninvasive assessment of the liver structure in their course is of crucial importance. The most accurate solution would be an invention of noninvasive parameters, obtained from the blood. Even though indirect and direct indices of liver fibrosis were found to be reliable and practical laboratory tools, new noninvasive markers in this field of hepatology are of crucial interest. Routinely obtained platelet (PLT) parameters (mean platelet volume (MPV), platelet distribution width (PDW), and plateletcrit (PCT)) are potential indicators of liver fibrosis. According to already performed surveys, MPV and PDW can be perceived as indicators of liver fibrosis in the course of HCV-related LC, correlating with serological concentration of direct markers of liver fibrosis and Fibroscan results [8]. Other researchers found a relationship between MPV and both: steatosis and liver fibrosis in patients diagnosed with HBV [9]. Of note, in another investigation, a decrease in MPV value within the HBV population of patients during the antiviral treatment was shown to be correlated with a regression of liver fibrosis [10]. Nevertheless, a very small number of already conducted studies compared diagnostic accuracy of hematological parameters with a clinical utility of indirect and direct indices of hepatic fibrosis. Subsequently, a potential role of hematological indices has been poorly explored in the course of liver steatosis. MPV describes the average PLT size, PDW is a measure of PLT size heterogeneity, and PCT shows percentage of blood occupied by platelets. A tight link between PLT parameters and liver disorders should be perceived as an interdisciplinary phenomenon. E.g., atherosclerosis, inflammatory bowel disease, malignancies, and cardiovascular complications constitute potential predisposing occurrences associated with deviations in PLT indices. Therefore, thrombotic events, hypersplenism, and the activation of bone marrow with coexisting inflammation in the liver parenchyma are key players involved in the disturbances of PLT markers [11–15]. To the best of our knowledge, the relationships between PLT indices and various serological markers of liver fibrosis have not been explored in ALC population, and data concerning NAFLD are limited. Moreover, the dependences between hematological markers and serological indices of liver fibrosis seem to be never investigated in Polish patients with liver disorders before. Therefore, we aimed to verify PLT indices in the course of ALC and NAFLD and to compare them with serological: direct and indirect markers of liver fibrosis. Another goal was to assess the relationship between PLT indices and clinical progression of liver failure in ALC.

## 2. Materials and Methods

The local ethics committee of the Medical University of Lublin approved the study (No. KE-0254/86/2016), and all patients signed an informed written consent in accordance with the Helsinki Declaration for the procedures they underwent.

### 2.1. Study Population and Research Design

**Figure 1:**
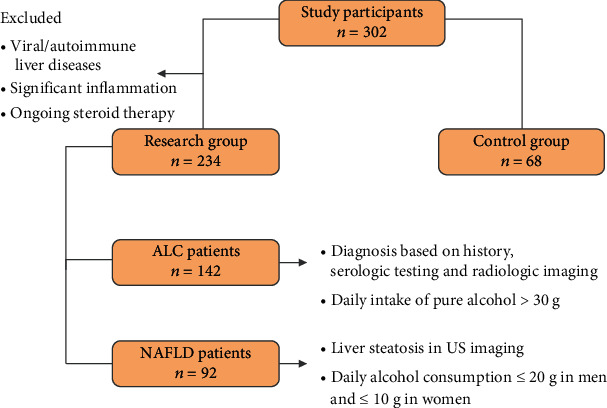
Flow chart displaying the selection of study participants. ALC: alcohol-related liver cirrhosis; NAFLD: nonalcoholic fatty liver disease; US: ultrasound.

Three hundred and two persons were retrospectively enrolled in the study: 142 patients with ALC, 92 with NAFLD, and 68 healthy volunteers in control group.  Figure 1 displays the selection of the participants included to the survey. The diagnosis of LC was based on history, serologic testing, and radiologic imaging. The liver biopsy was performed in 27 patients. In the remaining participants, the diagnosis of LC was based on clinical criteria and results of imaging studies, and liver biopsy was not required. The presence of hepatic encephalopathy and spontaneous bacterial peritonitis was excluded in the whole group. All participants included to the survey gained 0/9 points in CHESS (Clinical Hepatic Encephalopathy Staging Scale) scale. Alcoholic background of LC was diagnosed according to the proved daily intake of pure ethanol exceeding 30 g. A history of alcohol abuse was obtained directly from the patients or their family members. Moreover, all enrolled in the study ALC patients presented positive results of CAGE test. A diagnosis of NAFLD was established due to the history, physical examination, laboratory testing, and ultrasound imaging. A daily alcohol consumption did not exceed 20 g in men and 10 g in women. Certain diseases that can lead to steatosis (hepatobiliary infections, celiac disease, Wilson's disease, and alpha-1-antitrypsin deficiency) have been excluded. Twenty-two persons were diagnosed with diabetes mellitus type 2. People with diabetes mellitus type 1 were excluded from the study. None of the patients presented impaired fasting glucose. Forty-six NAFLD patients were found to have arterial hypertension, and metabolic syndrome was diagnosed in 84 persons. Viral and autoimmune liver disorders together with the presence of clinically significant inflammatory process were excluded in all participants. None of the persons included to the survey was on steroid therapy.

### 2.2. Procedures

Venous blood samples (peripheral blood) were collected from the studied patients and controls (S-Monovette, SARSTEDT, Aktiengesellschaft&Co., Nubrecht, Germany). Ethylenediaminetetraacetic acid (EDTA) was used to obtain hematological parameters and citrate to assess clotting indices. Biochemical markers were measured from the remaining blood sample without anticoagulant. The blood was obtained after at least 12 hours of fasting. Hematological and biochemical parameters were obtained 4 hours after blood sample collection. The analysis of morphotic blood indices was done with automatic ADVIA 2120i analyzer, Siemens and biochemical markers with ADVIA 1800 analyzer, Siemens. Prothrombin time (PT) and its International Normalized Ratio (INR) were measured with ACL TOP 500 analyzer, Instrumentation Laboratory. The part of blood samples without an anticoagulant was centrifuged at speed 2000 × g for 10 minutes within 15 minutes from blood collection. Obtained serum was stored in 1 ml Eppendorf test tubes in the temperature of -80°Celsius until the measurement of direct markers of liver fibrosis with enzyme-linked immunosorbent assay (ELISA). Among morphotic parameters of the blood, MPV, PCT, and PDW were obtained. The assessment of indirect indices of liver fibrosis included the following: AAR − AST/ALT (*AST* to *ALT* *Ratio*), APRI − ([AST/∗ULN]/PLT × [10^9^/l]) × 100; ∗*ULN - upper limit of normal* (*AST to PLT Ratio Index*), FIB − 4 − (age × AST/PLT × [10^9^/l]) × ALT^1/2^ (ang.*fibrosis* − 4), GPR − (GGT/ULN/PLT × [10^9^/l]) × 100 (*GGT* to *PLT* *Ratio*). MELD (model of end stage liver disease) score was used in ALC patients, and NAFLD fibrosis score and BARD score were used in the NAFLD group: MELD − 3.8[∗Ln bilirubin (mg/dl)] + 11.2[Ln INR] + 9.6[Ln creatinine (mg/dl)] + 6.4. ∗*Ln - natural logarithm*, NAFLD *fibrosis* *score* (NFS) − (−1.675) + 0.037 × age (years) + 0.094 × BMI (kg/m2) + 1.13 × impaired fasting glucose (IFG)/diabetes (YES − 1 point, NO − 0 points) + 0.99 × AST/ALT − 0.013 × PLT (×10^9^/l) − 0.66 × albumin (mg/dl), BARD *score* − AST/ALT ≥ 0.8 − 2 points, BMI ≥ 28 − 1 point; IFG/diabetes − 1 point; together 0-4 points. Among direct indices of liver fibrosis, procollagen I carboxyterminalpropeptide (PICP), procollagen III aminoterminalpropeptide (PIIINP), platelet-derived growth factor AB (PDGF-AB), transforming growth factor-*α* (TGF-*α*), and laminin were obtained. The measurement of PICP and PIIINP was performed with quantitative ELISA tests (Wuhan EIAab Science, Wuhan China). The measurement of PDGF-AB and TGF-*α* was done with R&D Systems Quantikine ELISA Kits (Minneapolis, MN, USA). Finally, the measurement of laminin was performed with Takara Laminin EIA Kit without sulphuric acid (Kusatsu, Shiga, Japan).

### 2.3. Statistical Analysis

Statistical analysis of the results was conducted using Statistica 13.0 (StatSoft Polska Sp. z o.o., Cracow, Poland) for Windows system. The demographic data and results of laboratory tests were presented as the mean value ± SD, and Student's *t*-test was used to compare these results. Deviation from normality was evaluated by Kolmogorov–Smirnov test. Data were expressed as the median and range (minimum–maximum). The Mann–Whitney *U* test was used for between-group comparisons because of nonnormal distribution. Spearman correlation analyses were used to verify the correlations. All probability values were two-tailed, and a value of *p* less than 0.05 was considered statistically significant. ROC (receiver operating characteristic) curves and AUC (area under the curve) values were applied to assess the sensitivity and specificity of examined markers and to evaluate proposed cutoffs of measured indices in the course of ALC and NAFLD.

## 3. Results

All ALC patients underwent esophagogastroduodenoscopy (EGD); in 126 persons, varices of the esophagus/stomach in the different stages were found. Ninety-two people were diagnosed with ascites, and 84 of them underwent paracentesis.  Table 1 presents characteristics of study participants.

### 3.1. Results of Scores, Hematological, and Serological Indices in Examined Study Participants

Table 2 shows results of used scores in the research group. Results of hematological parameters and serological (indirect and direct) indices of liver fibrosis are presented in Table 3.

Median MPV value in ALC patients was in a normal range; median PDW value turned out to be too high and PCT to be too low. Except median of MPV which was too low, medians of PDW and PCT in the NAFLD group were in a normal range. Median MPV value in ALC patients was the only one that did not differ significantly from the control group; median of PDW was significantly higher, and PCT was lower in comparison to controls. Medians of all obtained indices in the NAFLD group differed significantly from the control group. Medians of MPV and PCT were significantly lower, and PDW was higher in comparison to controls. The analysis of AAR, APRI, FIB-4, and GPR revealed their significantly higher medians in ALC patients compared to controls (*p* < 0.0001). Except for AAR, patients with NAFLD were found to have significantly higher values of all abovementioned indices in comparison to the control group (*p* < 0.0001). Among direct markers of liver fibrosis, median laminin value in the ALC group was significantly higher than in controls. Beside of PICP, medians of PIIINP, PDGF-AB, and TGF-*α* were significantly lower. Median values of TGF-*α* and laminin in NAFLD patients compared to controls turned out to be significantly lower.

### 3.2. Results of Correlations between Evaluated Markers in Examined Patients

Table 4 shows observed correlations between assessed markers in ALC and NAFLD patients.

PLT indices correlated positively with indirect indices of liver fibrosis in the ALC group: MPV with APRI, FIB-4, and GPR and PDW with APRI and FIB-4. There were also negative relationships between PCT and both: APRI and FIB-4. Strong positive correlations were noted between PCT and direct markers of liver fibrosis: PDGF-AB and TGF-*α*. MELD score correlated with PLT indices, positively with MPV and PDW and negatively with PCT. PDW correlated positively with indirect markers of liver fibrosis (APRI and FIB-4) and PCT, negatively, in NAFLD patients. There was a strong negative relationship between PCT and NFS and a weaker one between PCT and laminin. Finally, PDW and PCT correlated with each other negatively in ALC and NAFLD patients.

### 3.3. Diagnostic Accuracy of Investigated Parameters in Study Groups

Diagnostic accuracy of examined PLT indices is shown in Table 5.

**Figure 2:**
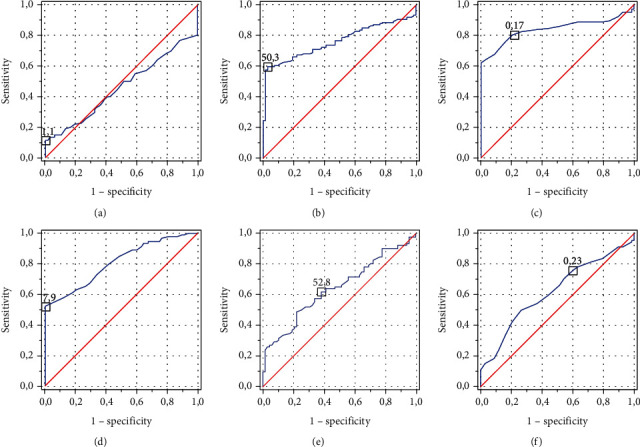
ROCs for assessed markers in observed patients: (a) MPV in the ALC group; (b) PDW in the ALC group; (c) PCT in the ALC group; (d) MPV in the NAFLD group; (e) PDW in the NAFLD group; (f) PCT in the NAFLD group.

ROCs presenting examined platelet indices in ALC and NAFLD patients are presented in Figure 2. AUC values and proposed cutoffs for MPV, PDW, and PCT in ALC patients were 0.458 (>11.1 fl), 0.764 (>59.3%), and 0.839 (<0.17%), respectively. AUC values and proposed cutoffs for MPV, PDW, and PCT in NAFLD patients were 0.808 (<7.9 fl), 0.643 (>52.8%), and 0.622 (<0.23%), respectively.

## 4. Discussion

Platelet indices were shown to participate in the pathological appearance of cardiovascular diseases, cancer, or stroke. However, the number of studies devoted to possible linkage between hematological parameters and indirect and direct markers of liver fibrosis is not satisfactory, and the role of MPV, PDW, and PCT in liver diseases still remains unclear. Inflammatory process, splenomegaly, and secondary activation of the bone marrow are potential mechanisms responsible for the deviations in PLT indices in patients with liver disorders. Enhanced breakdown of PLTs due to hypersplenism together with increased release of interleukin-6 shortens PLT life cycle. In the consequence, the production of PLTs by the bone marrow rises, promoting the release of larger, reticulated PLTs into the bloodstream. Theoretically, aforementioned disturbances should be reflected by an increase in MPV, PCT, and PDW. According to available literature, higher level of MPV might be even perceived as a prognostic factor of advanced fibrosis in primary biliary cholangitis patients, marker of hepatocellular carcinoma, and a parameter predisposing to the transformation of simple steatosis into steatohepatitis in the course of NAFLD [16–20]. The elevation in MPV among patients with liver failure has been also suggested as an inflammatory marker due to the consumption of large active PLTs in the course of the disease [21–24]. Additionally, higher levels of MPV were proposed by Adeles et al. as predictors of cardiovascular complications in NAFLD patients [25]. Our results proved previous observations in the ALC group; MPV correlated positively with MELD score. Unexpectedly, a significant decrease in MPV turned out to be a quite powerful marker in the course of NAFLD (AUC = 0.808). Higher values of PDW and PCT seem to accompany advanced liver fibrosis in HBV- and HCV-related chronic hepatitis. Moreover, PCT was even described as a positive prognostic marker in the early detection of NAFLD [26–28]. In contrast, Coskun with collaborators found its low level to be associated with advanced fibrosis in HCV-related cirrhosis [29]. Of note, Wang et al. confirmed this dependency among HBV patients, recently. PCT in this survey was a parameter predicting significant or advanced fibrosis and cirrhosis with a quite good diagnostic accuracy (AUC = 0.645, AUC = 0.709, and AUC = 0.714, respectively). Moreover, AUC value of PCT was greater compared to APRI for predicting advanced fibrosis and cirrhosis (AUC = 0.638 and AUC = 0.637, respectively) [30]. Our results support this point of view; a decrease in PCT level turned out to have the greatest diagnostic accuracy in our ALC patients (AUC = 0.839); its level correlated negatively with MELD score. A little of available data concern the role of PDW and PCT in the population of patients with ALC. A great majority of surveys is devoted to HBV- and HCV-related liver cirrhosis. A recent observation done by Shao et al. revealed significant correlations between PLT indices (MPV and PDW) and direct indices of liver fibrosis (PIIINP, collagen IV, and laminin) in HCV-infected patients. More marked increase in MPV was associated with more advanced liver fibrosis in this group of persons [8]. On the contrary, another study group did not find MPV to be a sufficient parameter in predicting more advanced fibrosis stages in HCV patients [31]. Recently, Ramadori and colleagues have highlighted a wide spectrum of potential interactions between PLTs and liver cells. A tight junction between PLT activity and different stages of liver pathologies appears to be a promising pathway even in the treatment of liver disorders [32]. We noticed strong positive dependences between PCT and both: PDGF-AB and TGF-*α* in the ALC group and a negative one between PCT and laminin in NAFLD patients; to the best of our knowledge, these are the first findings reported in the course of CLDs, so far. The goal of our survey was not to compare a diagnostic accuracy of selected hematological indices between ALC and NAFLD patients. We tried to figure out whether an isolated liver steatosis might be affected by certain deviations in hematological indices. Our study evaluated the population of patients with NAFLD without the assessment of coexisting hepatitis in liver biopsy. A further direction should concern the differentiation of the patients with a simple steatosis and steatohepatitis in the context of hematological indices. It is worth highlighting the involvement of PLT markers in the course of portal hypertension (PH), as well. More and more available surveys are invented nowadays to reveal a relationship between PLTs, PLT indices in combination with various laboratory/imaging techniques, and the presence of esophageal varices (EVs) in cirrhotic patients [33]. A decreased value of PLTs (implemented recently in Liaoning Score with a formula based on the presence of acute upper gastrointestinal bleeding (AUGIB), ascites, and PLT count) is believed to be such a parameter. The first survey devoted to Liaoning score proved that ascites and PLTs in the course of LC are independent predictors for EVs in patients with AUGIB. However, in the group without AUGIB, PLTs were shown to be the single predictor for EVs [34]. Consequently, the study by Qianqian et al. on cirrhotic patients revealed that AUC of Liaoning score for predicting EVs was greater compared to APRI, AAR, and FIB-4 (0.737 vs. 0.650, 0.626, and 0.709, respectively) [35]. Other investigations suggested that in patients who do not meet Baveno VI criteria, the extension with PLT/liver stiffness measurement (Fibroscan) ratio makes it possible to avoid EGD in this population (AUC = 0.726) [36]. On the other hand, PLT count was the marker helpful in identifying patients without high-risk varices in HBV-related compensated cirrhosis patients who did not meet Baveno VI criteria [37]. We did not try to figure out if there is any association between the presence of PH (ascites, EVs) and PLT count together with abnormalities in PLT indices among ALC patients included to the current survey. It was not the aim of our research; however, a further evaluation of these dependences may be very interesting, and this issue will constitute the subject of our new study. Similarly, noninvasive serum liver fibrosis indexes were also proposed as potential markers with an overall modest to low diagnostic accuracy for PH [38]. The comparison of the measurement of PH with hepatic venous pressure gradient with indirect markers of liver fibrosis revealed that AUC of APRI for predicting clinically significant PH and severe PH was 0.740 and 0.742, respectively [39]. Ahmed et al. presented recently that PLTs, spleen area, and APRI score (AUC = 0.846, AUC = 0.828, and AUC -0.827, respectively) might be perceived as quite reliable markers of PH [40]. Another comparison between the utility of acoustic radiation force impulse imaging and APRI in the assessment of PH revealed their similar diagnostic accuracy (AUC = 0.855 and AUC = 0.838, respectively) [41]. APRI, AAR, and FIB-4 were also evaluated as potential markers in the evaluation of the presence of EV and their bleeding. Only FIB-4 and APRI turned out to be significant predictors of variceal bleeding [42]. We did not exam the relationship between the presence of EVs and indirect markers of liver fibrosis in our population of ALC patients. Thus, it might be assumed that this topic requires further investigations, and a certain role of noninvasive markers in the diagnosis of PH should be explored.

## 5. Conclusions

A current survey revealed that PLT indices can be treated as potential prognostic markers in ALC and NAFLD patients, correlating significantly with MELD score, serological markers of liver fibrosis, and NFS. According to our observations, PCT in ALC and MPV in NAFLD turned out to be the most powerful markers.

## Figures and Tables

**Table 1:** Clinical characteristics of study participants.

Parameter	ALC *n* = 142	NAFLD *n* = 92	Controls *n* = 68	Together *n* = 302
Sex (w/m)	36/106	33/59	36/32	105/197
Age (years) (*x* ± *s*; me; min-max)	54 ± 12; 55; 31-84	60 ± 15; 61; 22-90	46 ± 16; 45; 20-85	54 ± 15; 55; 20-90
BMI (kg/m^2^) (*x* ± *s*; me; min-max)	25.89 ± 9.31; 25.91; 16.7-36.71	29.49 ± 4.9; 28.7; 16.26-43.01	21.95 ± 2.62; 22.45; 16.18-24.86	—
DM 2	0/142	22/92	—	—
AH	32/142	46/92	—	—

w: women; m: men; *x*: mean; *s*: standard deviation; me: median; min: minimum; max: maximum; BMI: body mass index; DM: diabetes mellitus; AH: arterial hypertension.

**Table 2:** Results of used scores in research group.

Score	ALC					NAFLD				
	*x*	*s*	me	min	max	*x*	*s*	me	min	max
MELD	17	8	16	6	45	—	—	—	—	—
BARD score	—	—	—	—	—	2	1	2	0	4
NAFLD fibrosis score	—	—	—	—	—	-1.36	1.5	-1.16	-5.83	1.74

**Table 3:** Results of hematological indices and serological (indirect and indirect) markers of liver fibrosis in examined patients.

Parameter (reference range)	ALC					NAFLD					Controls				
	*x*	*s*	me	min	max	*x*	*s*	me	min	max	*x*	*s*	me	min	max
PLT (130 − 400 × 10^9^/l)	119	81	93^∗∗∗∗^	4	459	248	78	230^∗∗∗^	8	506	293	62	297	160	400
MPV (8-11 fl)	9.12	1.51	8.8	6.6	14.4	7.94	0.95	7.85^∗∗∗∗^	6.3	10.5	9.09	0.88	8.90	8	11
PDW (40-60%)	58.87	11.27	60.4^∗∗∗∗^	20.4	95.9	54.91	7.98	54.75^∗∗^	38	74.5	51.08	5.27	51.2	40.7	59.8
PCT (0.12-0.3%)	0.13	0.09	0.1^∗∗∗∗^	0	0.57	0.2	0.08	0.19^∗∗^	0.06	0.63	0.22	0.05	0.21	0.13	0.3
AAR	2.19	1.16	1.89^∗∗∗∗^	0.18	7.57	1.03	0.55	0.91^∗^	0.23	3.1	1.15	0.43	1.1	0.43	2.86
APRI	4.35	7.02	2.43^∗∗∗∗^	0.15	68.38	0.81	1.04	0.48^∗∗∗∗^	0.13	7.67	0.25	0.13	0.23	0.11	0.86
FIB-4	11.67	25.46	6.34^∗∗∗∗^	0.69	287.59	1.92	1.63	1.57^∗∗∗∗^	0.23	11.58	0.85	0.54	0.71	0.28	3.27
GPR	15.73	28.54	6.65^∗∗∗∗^	0.18	188.71	2.76	5.57	0.54^∗∗∗∗^	0.13	35.41	0.25	0.1	0.24	0.06	0.63
PICP (ng/ml)	63.32	31.53	60.53	6.15	161.12	52.14	27.56	46.08	10.10	147.27	58.26	37.39	44.18	0	202.89
PIIINP (ng/ml)	9.28	4.33	8.4^∗∗^	2.43	28.65	11.41	3.99	11.00	2.18	25.35	11.07	5.61	10.25	4.35	43.63
PDGF-AB (pg/ml)	18280.47	806.06	17343.71^∗∗∗^	1925.68	42823.84	26858.68	7335.09	26682.83	10821.02	49808.07	23579.28	10068.8	25623.2	1638.2	47758.7
TGF-*α* (pg/ml)	24	45.33	13.77^∗∗∗∗^	0.872	507.09	17.89	19.18	12.09^∗∗∗∗^	1.39	142.63	28.44	17.21	24.59	1.31	93.55
Laminin (ng/ml)	976.34	705.29	832.06^∗^	101.933	3301.00	48	230.24	375.23^∗∗∗∗^	72.87	1335.92	718.24	386.1	663.27	140.88	1813.88

^∗^ *p* < 0.05, ^∗∗^*p* < 0.01, ^∗∗∗^*p* < 0.001, ^∗∗∗∗^*p* < 0.0001.

**Table 4:** Correlations between examined parameters in examined ALC and NAFLD patients.

Pair	*R* spearman	*p*
ALC		
PDW and PCT	-0.193	^∗^
MPV and APRI	0.300	^∗∗∗^
MPV and FIB-4	0.299	^∗∗∗^
MPV and GPR	0.399	^∗∗∗^
PDW and APRI	0.334	^∗∗∗∗^
PDW and FIB-4	0.373	^∗∗∗∗^
PDW and GPR	0.312	^∗∗∗^
PCT and APRI	-0.483	^∗∗∗∗^
PCT and FIB-4	-0.585	^∗∗∗∗^
MPV and MELD	0.306	^∗∗∗^
PDW and MELD	0.310	^∗∗∗^
PCT and MELD	-0.186	^∗^
PCT and PDGF-AB	0.386	^∗∗∗^
PCT and TGF-*α*	0.208	^∗^
NAFLD		
PDW and PCT	-0.312	^∗∗^
PDW and APRI	0.267	^∗^
PDW and FIB-4	0.264	^∗^
PCT and APRI	-0.330	^∗∗^
PCT and FIB-4	-0.464	^∗∗∗∗^
PCT and NFS	-0.516	^∗∗∗∗^
PCT and laminin	0.242	^∗^

^∗^ *p* < 0.05, ^∗∗^*p* < 0.01, ^∗∗∗^p < 0.001, ^∗∗∗∗^p < 0.0001. NFS: NAFLD fibrosis score.

**Table 5:** Diagnostic accuracy of examined indices in ALC and NAFLD patients.

Parameter	ALC						NAFLD					
	Diagnostic accuracy						Diagnostic accuracy					
	AUC	Sensitivity	Specificity	PPV	NPV	*p*	AUC	Sensitivity	Specificity	PPV	NPV	*p*
MPV	0.458	11%	100%	100%	35%	—	0.808	52%	100%	100%	61%	^∗∗∗∗^
PDW	0.764	60%	97%	98%	54%	^∗∗∗∗^	0.643	62%	62%	69%	55%	^∗∗∗^
PCT	0.839	80%	79%	89%	66%	^∗∗∗∗^	0.622	76%	40%	63%	55%	^∗∗^

^∗^ *p* < 0.05, ^∗∗^*p* < 0.01, ^∗∗∗^*p* < 0.001, ^∗∗∗∗^*p* < 0.0001. AUC: area under the curve; PPV: positive predictive value; NPV: negative predictive value.

## Data Availability

The data is available in the supplemental files and upon corresponding author request.

## Supplementary Materials

Supplementary 1 1: NAFLD: the description of results in NAFLD group.

Supplementary 2 2: ALC vs controls: a comparison between ALC and controls.

Supplementary 3 3: ALC: the description of results in ALC group.
