# Supplementary figures and images for: Plateletcrit and Mean Platelet Volume in the Evaluation of Alcoholic Liver Cirrhosis and Nonalcoholic Fatty Liver Disease Patients
Source: Biomed Res Int. 2021 Feb 15;2021:8867985. doi: 10.1155/2021/8867985 (PMC7901043; doi:10.1155/2021/8867985)

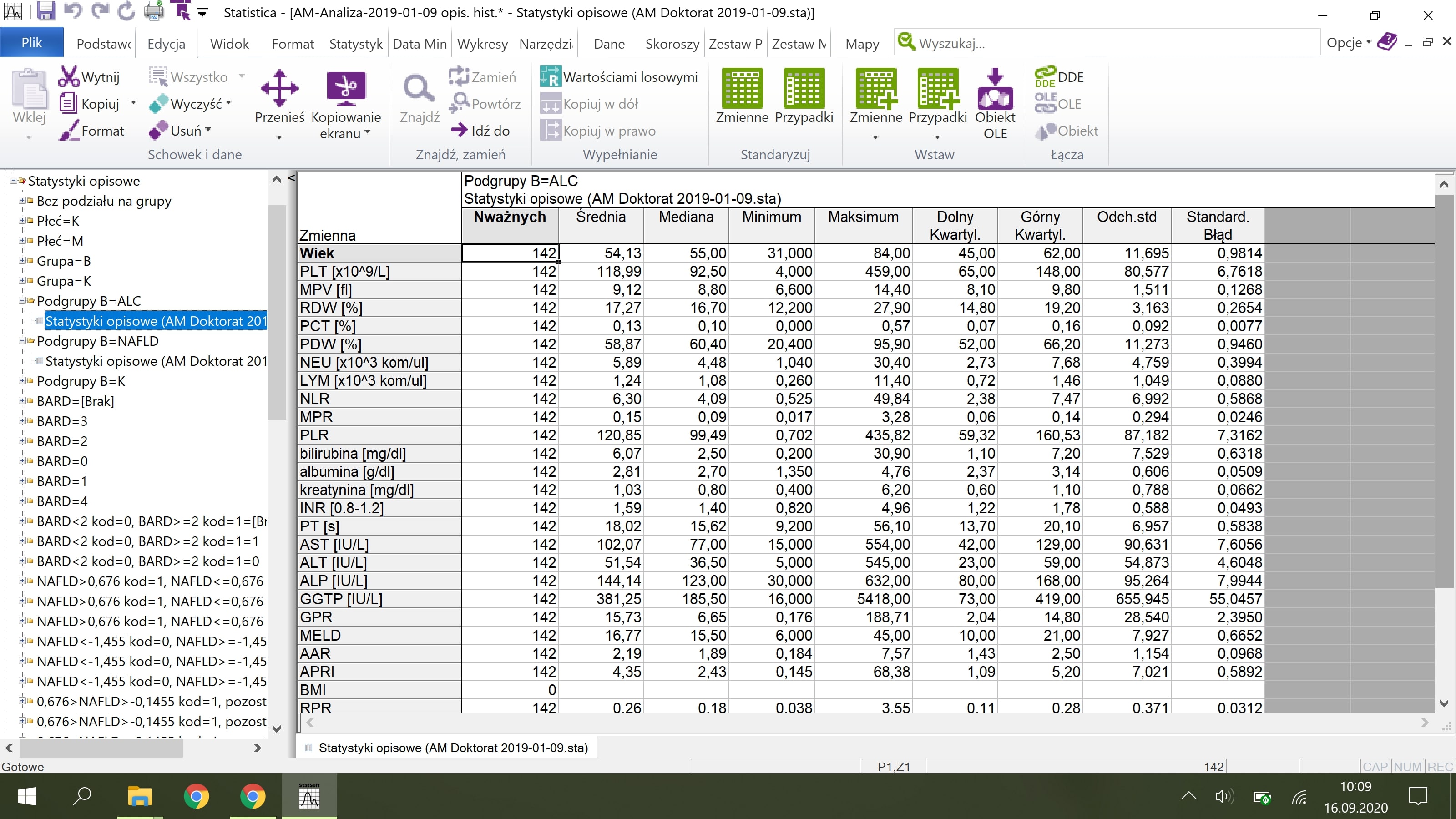

Supplement: Supplementary 1 — 1: NAFLD: the description of results in NAFLD group. [file 8867985.f1.jpg]

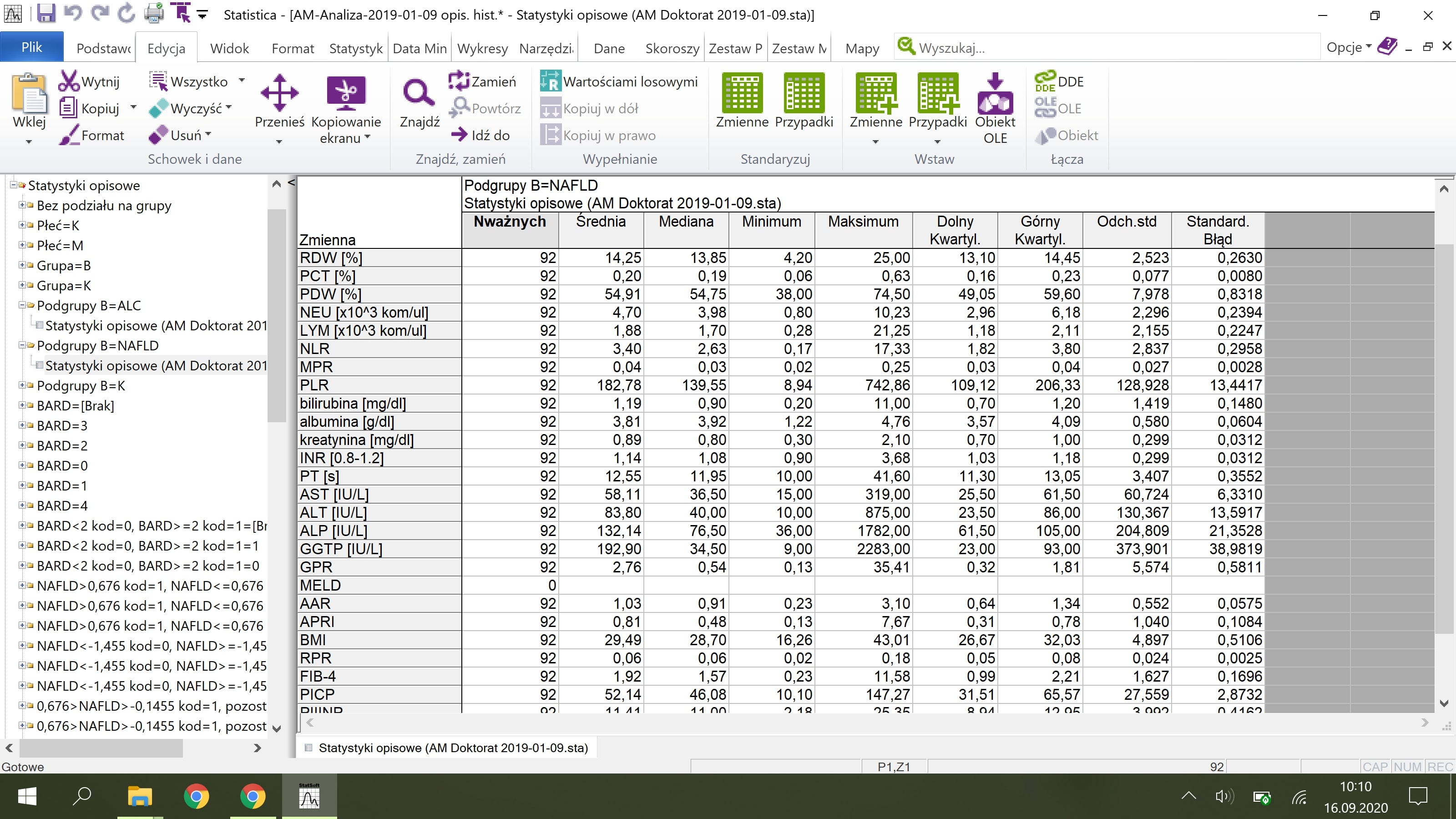

Supplement: Supplementary 2 — 2: ALC vs controls: a comparison between ALC and controls. [file 8867985.f2.jpg]

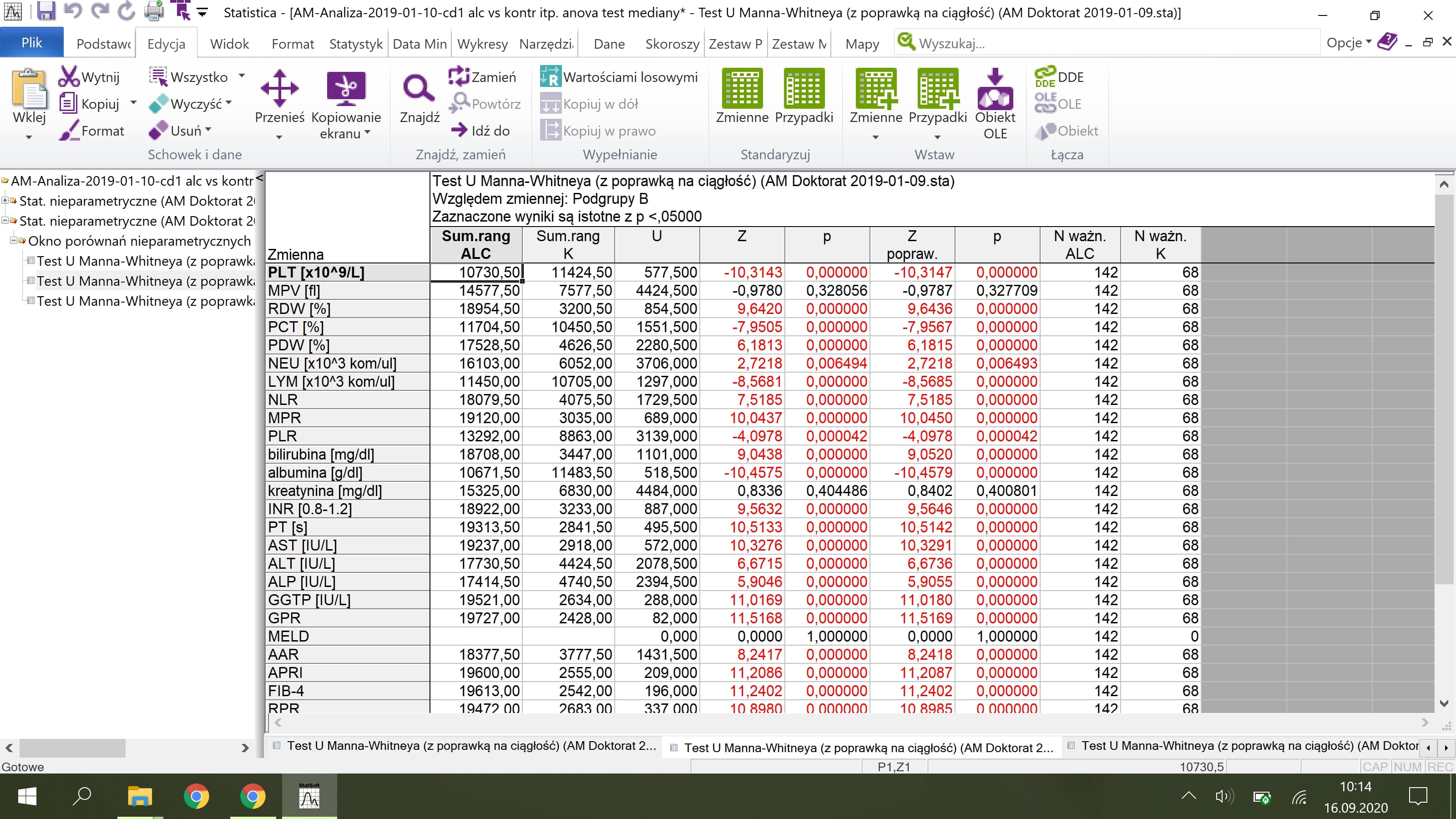

Supplement: Supplementary 3 — 3: ALC: the description of results in ALC group. [file 8867985.f3.jpg]
